# Supplementary material for: Oxygen level alters energy metabolism in bovine preimplantation embryos
Source: Sci Rep. 2025 Apr 2;15:11327. doi: 10.1038/s41598-025-95990-z (PMC11965477; doi:10.1038/s41598-025-95990-z)
Supplement: Supplementary file 1 — Supplementary Material 1 [file 41598_2025_95990_MOESM1_ESM.pdf]

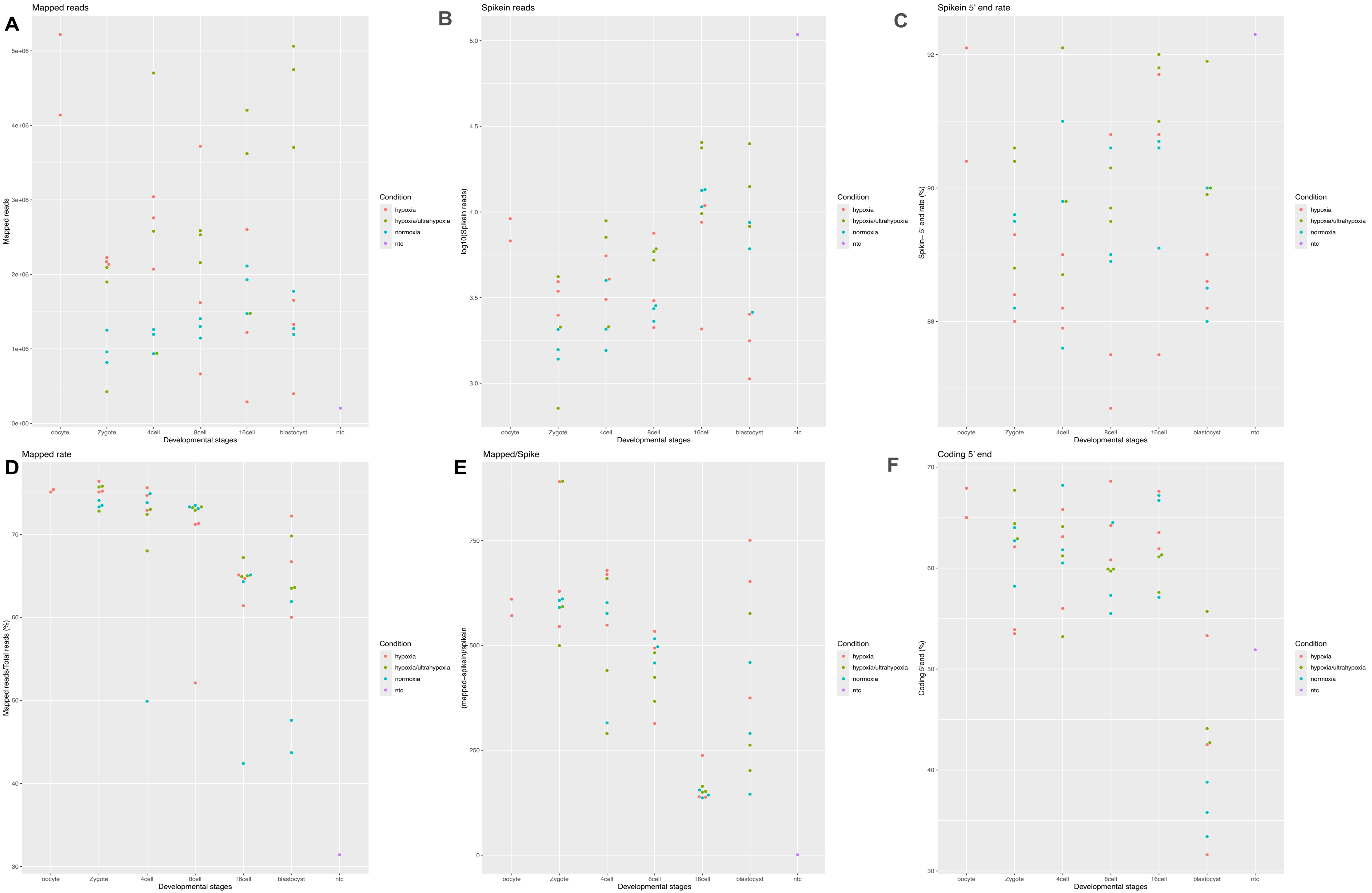

Supplementary Figure 1. Quality control analysis of the STRT-N RNA sequencing library.
A) Graph showing total number of mapped reads. B) Total number of ERCC Spike- in reads per sample. C) Quality check of the 5'-end spike-in rate. D) Mapped rate calculated by dividing the total number of reads with total number of mapped reads. E) Mapped/Spike-in reads, a quality check of the overall amount of mRNA present in each sample. F) Coding 5'-end of the mRNA molecules. On the x-axis of all the samples we have developmental stages. Hypoxia is shown in red, normoxia in blue and ultrahypoxia in green.
