# Supplementary material for: Oxygen level alters energy metabolism in bovine preimplantation embryos
Source: Sci Rep. 2025 Apr 2;15:11327. doi: 10.1038/s41598-025-95990-z (PMC11965477; doi:10.1038/s41598-025-95990-z)
Supplement: Supplementary file 2 — Supplementary Material 2 [file 41598_2025_95990_MOESM2_ESM.pdf]

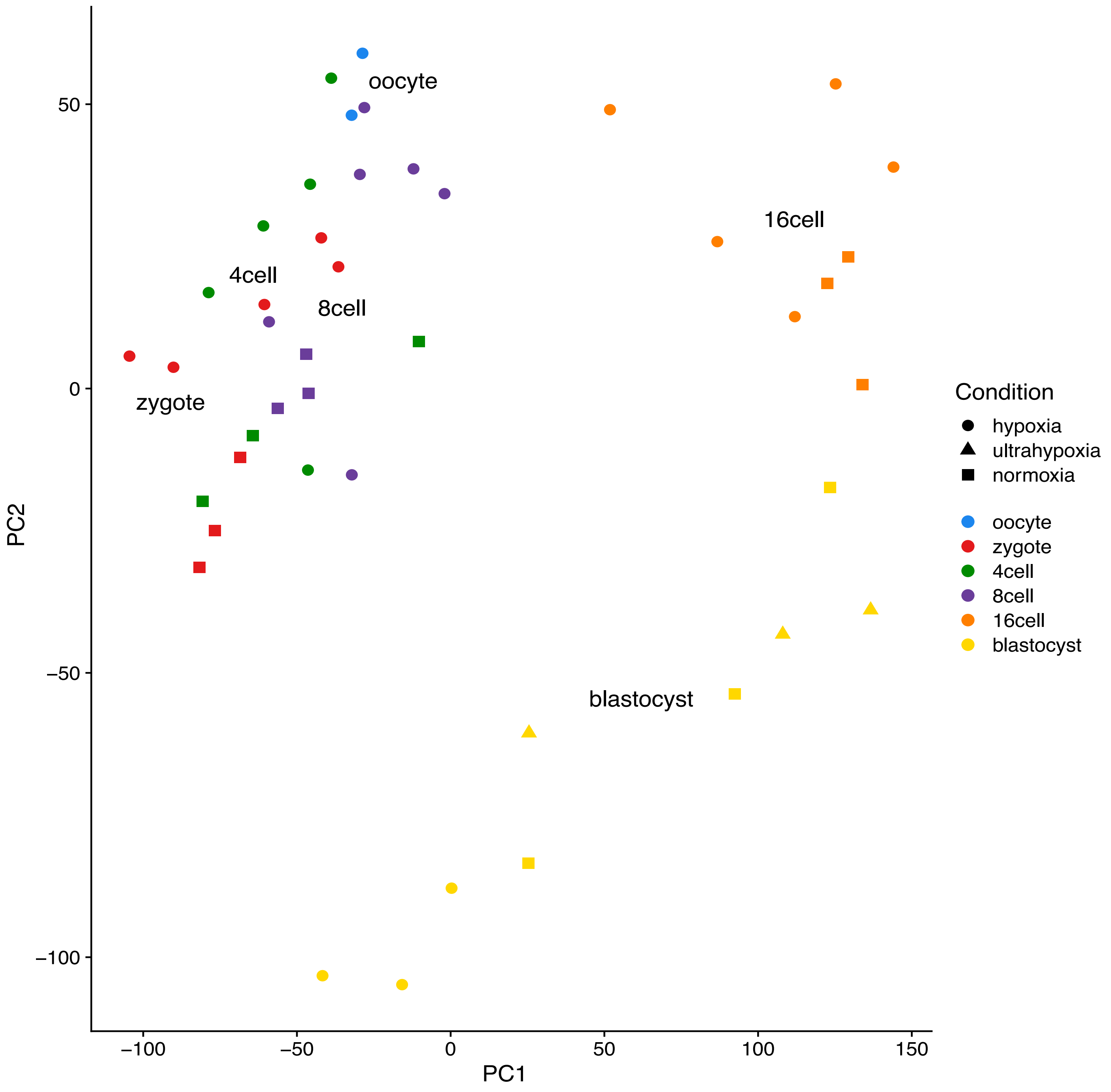

Supplementary Figure 2. Principal component analysis.
Principal component analysis with all the samples used for the downstream analysis. PC1 shows the biggest variance between samples according to the developmental stages of the embryo development, separating the earlier stages of development: oocytes, zygotes, 4- and 8-cell embryos; from the later stages: 16-cell embryos and blastocysts. PC2 separates blastocysts from the earlier stages of embryo development.
