# Supplementary material for: Oxygen level alters energy metabolism in bovine preimplantation embryos
Source: Sci Rep. 2025 Apr 2;15:11327. doi: 10.1038/s41598-025-95990-z (PMC11965477; doi:10.1038/s41598-025-95990-z)
Supplement: Supplementary file 3 — Supplementary Material 3 [file 41598_2025_95990_MOESM3_ESM.pdf]

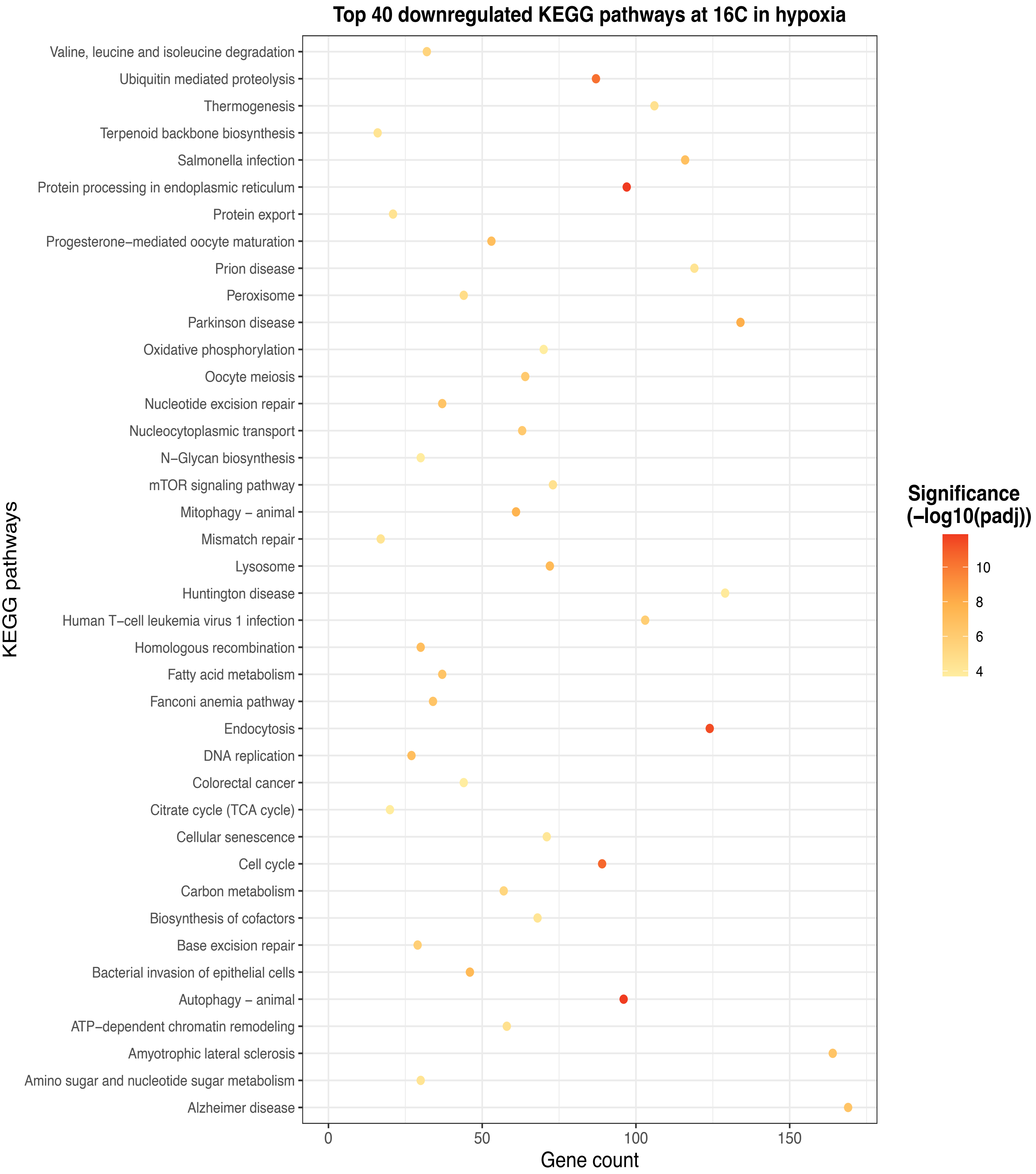

Supplementary Figure 3. TOP 40 downregulated KEGG pathways in 16-cell embryos in hypoxia.
Top 40 KEGG pathways separated based on their p. adjusted value can be shown on the y-axis. On the x-axis the count of genes involved in the pathways can be seen, while the color of the circle is based on the significance value.
