# Supplementary material for: Oxygen level alters energy metabolism in bovine preimplantation embryos
Source: Sci Rep. 2025 Apr 2;15:11327. doi: 10.1038/s41598-025-95990-z (PMC11965477; doi:10.1038/s41598-025-95990-z)
Supplement: Supplementary file 6 — Supplementary Material 6 [file 41598_2025_95990_MOESM6_ESM.docx]

Supplementary table 2A. Cleavage and blastocyst rate in normoxia. Blastocyst rate calculated based on the number of presumptive zygotes.

| **N**  **O**  **R**  **M**  **O**  **X**  **I**  **A** | **Number of presumptive zygotes** | **Number of cleaved embryos** | **Number of blastocysts d7** | **Number of blastocysts d8** |
| --- | --- | --- | --- | --- |
|  | 50 | 41 | 1 | 4 |
|  | 50 | 44 | 7 | 9 |
|  | **100** | **85%** | **8%** | **13%** |

Supplementary table 2B. Cleavage and blastocyst rate in hypoxia. Blastocyst rate calculated based on the number of presumptive zygotes.

| **H**  **Y**  **P**  **O**  **X**  **I**  **A** | **Number of presumptive zygotes** | **Number of cleaved embryos** | **Number of blastocysts d7** | **Number of blastocysts d8** |
| --- | --- | --- | --- | --- |
|  | 50 | 43 | 15 | 18 |
|  | 50 | 42 | 17 | 19 |
|  | 50 | 43 | 17 | 17 |
|  | 14 | 13 | 5 | 5 |
|  | **164** | 85.97% = **86%** | 32.92%= **33%** | 35.97%= **36%** |

Supplementary table 2C. Cleavage and blastocyst rate in ultrahypoxia. Blastocyst rate calculated based on the number of presumptive zygotes.

| **ULTRA**  **H**  **Y**  **P**  **O**  **X**  **I**  **A** | **Number of presumptive zygotes** | **Number of cleaved embryos** | **Number of blastocysts d7** | **Number of blastocysts d8** |
| --- | --- | --- | --- | --- |
|  | 50 | 35 | 1 | 1 |
|  | 50 | 42 | 1 | 4 |
|  | 50 | 38 | 0 | 0 |
|  | 44 | 38 | 2 | 4 |
|  | **194** | 78.86%= **78.9%** | 2.06%= **2.1%** | 4.63%= **4.6%** |
